# A Multilevel Analysis of Absence of Transport to a Hospital Before Premature Cardiac Death

**Published:** 2010-04-15

**Authors:** Elizabeth Barnett Pathak, Michele L. Casper, Jean Paul Tanner, Steven Reader, Beverly Ward

**Affiliations:** Department of Epidemiology and Biostatistics, College of Public Health, University of South Florida; Centers for Disease Control and Prevention, Atlanta, Georgia; University of South Florida, Tampa, Florida; University of South Florida, Tampa, Florida; University of South Florida, Tampa, Florida

## Abstract

**Introduction:**

Prompt transportation to a hospital and aggressive medical treatment can often prevent acute cardiac events from becoming fatal. Consequently, lack of transport before death may represent lost opportunities for life-saving interventions. We investigated the effect of individual characteristics (age, sex, race/ethnicity, education, and marital status) and small-area factors (population density and social cohesion) on the probability of premature cardiac decedents dying without transport to a hospital.

**Methods:**

We analyzed death data for adults aged 25 to 69 years who resided in the Tampa, Florida, metropolitan statistical area and died from an acute cardiac event from 1998 through 2002 (N = 2,570). Geocoding of decedent addresses allowed the use of multilevel (hierarchical) logistic regression models for analysis.

**Results:**

The strongest predictor of dying without transport was being unmarried (odds ratio, 2.13; 95% confidence interval, 1.79-2.52, *P* < .001). There was no effect of education; however, white race was modestly predictive of dying without transport. Younger decedent age was a strong predictor. Multilevel statistical modeling revealed that less than 1% of the variance in our data was found at the small-area level.

**Conclusions:**

Results contradicted our hypothesis that small-area characteristics would increase the probability of cardiac patients receiving transport before death. Instead we found that being unmarried, a proxy of living alone and perhaps low social support, was the most important predictor of people who died from a cardiac event dying without transport to a hospital.

## Introduction

Premature deaths from heart disease usually result from a severe, acute event such as an acute myocardial infarction (AMI) ("heart attack") or, less commonly, a sudden cardiac arrest (SCA). In most cases, the onset of these life-threatening events begins at home (1). Prompt transportation to a hospital and aggressive medical care can often prevent death in these severe cases; however, many victims die at home without initiating an attempt at transport to a hospital (1). Transportation to a hospital in the United States occurs both by professional emergency medical services (EMS) and by self-transport (usually by private motor vehicle). It is not uncommon for people with AMI to attempt to drive themselves to the nearest hospital.

In the United States, as in many industrialized nations, most decedents die in a hospital (2). This situation reflects both the extensiveness of medical services and intensiveness of medical interventions for chronic diseases at the end of life. Consequently, we can view deaths from cardiac events that occur before transport as a result of 3 general scenarios: 1) onset of cardiac symptoms is severe and death follows rapidly, with no time to solicit or initiate transportation (eg, sudden cardiac death); 2) the patient delays or avoids seeking medical treatment and death follows at some point hours later; 3) the patient desires transport to a hospital but is unable to access transportation before death. Under the last scenario, the patient’s inability to access transportation could result from several factors, including lack of family member, neighbor, or bystander support for communication and transportation; lack of telephone; lack of vehicle for personal transportation; lack of local availability of EMS; being a long distance from the nearest hospital; and lack of insurance or financial means to obtain transportation.

Many, if not most, people experiencing cardiac symptoms delay seeking medical treatment (1). Early professional approaches to this problem assumed that lack of knowledge of heart attack symptoms was the source of patient delays. However, more recent research has demonstrated that lack of knowledge is rarely the most important reason for patient delays in seeking treatment (1). Rather, reasons for delays in treatment seeking are complex and relate to patients' age, sex, cultural and racial/ethnic background, socioeconomic status, insurance coverage, geographic location, medical history, availability of social support, and cognitive and emotional factors (1).

We have previously described how "no transport" deaths from cardiac events may represent lost opportunities for life-saving medical intervention (3). Although SCAs are rapid-onset events that require immediate medical intervention to ensure resuscitation and reperfusion of the heart (4), the typical clinical development of an AMI opens a wider time frame for medical intervention (5). Both individual (patient) factors (1,3,6) and local social environmental (small-area) factors (3,7,8) may influence the use of medical care resources during an acute cardiac event. Availability of an acute-care hospital and local EMS are essential requirements, but even in areas with reasonable availability of services, many deaths from cardiac events occur without transport (8).

On the basis of findings from previous research, we hypothesized that both sociodemographic characteristics of decedents (unmarried, male, younger age, white race, and lower educational attainment) and social environmental characteristics of small areas (low population density and low social cohesion) would increase the probability that people would die from a cardiac event without transport to a hospital. Social cohesion is an ecologic construct that attempts to capture the overall extent of social ties and connectedness in a defined community. Low community social cohesion has been independently associated with heart disease risk (9,10). We focused on premature deaths because complex factors influence the desirability of hospitalization and aggressive medical intervention among elderly patients, particularly the very elderly (2). In contrast, for nonelderly persons who experience cardiac events, there exists a social and cultural presumption about the importance and necessity of medical intervention in industrialized countries such as the United States (2).

## Methods

### Study population and definitions

Our study population consisted of adults aged 25-69 years who died from an acute cardiac event during 1998 through 2002 in Tampa, Florida. We ascertained cardiac decedent status from death certificates. An acute cardiac death was defined as any death for which the coded underlying cause was AMI (International Classification of Diseases, Ninth Revision [ICD-9] codes 410-411; ICD-10 code I21), cardiac arrest/cardiac dysrthymias (ICD-9 codes 427.4, 427.5, 427.9; ICD-10 codes I46.1, I46.9, I49.0, I49.9), cardiovascular disease unspecified (ICD-9 code 429.2; ICD-10 code I51.6), or ill-defined and unknown causes (ICD-9 codes 798.1, 798.9, 799.1, 799.9; ICD-10 codes R09.2, R57.0, R96.0, R98, R99). We included cardiovascular disease unspecified because under ICD-9, this code was used for cases of myocardial infarction and ischemic heart disease with insufficient diagnostic information (11). Because the likelihood of misclassification of cause-of-death coding for heart disease is not independent of place of death (the basis of our main study outcome), we used a definition of acute cardiac deaths that included definite, probable, and possible AMIs and sudden cardiac deaths.

The cause-of-death category ill-defined and unknown causes (ID) is used when postmortem evidence is insufficient to support assigning a specific disease as cause of death (12). Earlier research on SCA fatalities indicated that these were often coded as ID on the death certificate (13). A study from Belgium found that approximately 5% of definite or possible cases of AMI had been coded as ID on the death certificate (11).

Our outcome in this study was no transport before cardiac death. We obtained transport status information for each decedent from the place of death variable on the death certificate. We categorized a cardiac death as occurring with no transport if the place of death was reported as either at home or in another location in the community. Deaths that occurred during or after transport had place of death reported as 1 of the following: 1) dead on arrival (at hospital), 2) emergency room/outpatient, 3) hospital inpatient, or 4) hospital, unknown inpatient or outpatient status. Variables we examined were age by group (25-49, 50-59, 60-64, and 65-69 years), sex, race/ethnicity (non-Hispanic whites vs Hispanics and non-Hispanic blacks), educational attainment (no college degree vs college degree) and marital status (unmarried vs married).

### Small-area variables

Our study area was the Tampa-St. Petersburg-Clearwater metropolitan statistical area (Tampa MSA). This ethnically and geographically diverse area of more than 2 million people encompasses 2,600 square miles of central cities, suburbs, small towns, and rural farms spread across 4 counties. Our geographic unit of analysis was the public use microdata area (PUMA) (14). Population and housing data for the 20 PUMAs in our study area were obtained from the public use microdata 5% sample from the 2000 census (14).

We first geocoded each decedent record by using the address of residence from the death certificate, using the geocoding utility in ESRI ArcGIS (ESRI, Redlands, California), supplemented by manual geocoding for all cases without an exact address match. The ESRI ArcGIS street database was our reference for geocoding, supplemented by zip code and street maps of the area. We matched 97% of decedent records to the street address level. None of the decedents were geocoded to zip code centroids or other proximal locations.

We examined 2 dimensions of the social environment that we hypothesized would influence access to and use of transportation (whether private or EMS). The first was low population density. We looked at 3 indicators: land area of the PUMA in square miles, percentage of all housing units that were sited on 1 or more acres of land, and percentage of all housing units that were detached single-family homes (as opposed to apartment buildings, duplexes, and other types of dense housing). The second dimension was low social cohesion. We looked at 3 indicators for the population aged 25 to 69 years: percentage who lived alone, percentage who had resided at the same address for less than 5 years, and percentage who had resided at the same address for more than 10 years.

### Analytic methods

We fit multilevel random effects logistic regression models to our data to quantify the independent effects of social environmental (PUMA level) and decedent (individual level) predictor variables on the probability of a cardiac decedent dying before transport to a hospital. We used the logit link in the June 2006 release of the GLIMMIX procedure in SAS version 9.1.3 (SAS Institute, Inc, Cary, North Carolina) for statistical analyses (15,16). Following a typical multilevel modeling strategy (17), we first fit a null model with no fixed effects and random intercept effects for the 20 small areas:

Equation 1

log [*p_ij_* / (1 − *p_ij_*)] = β_*0*_ + *u_j_*

where *p_ij_* is the probability of the *i*th individual in the *j*th PUMA dying before transport, and *u_j_* is the random effect at the PUMA level. Then we fit separate models for each of the social environmental predictor variables, each with the following form:

Equation 2

log [*p_ij_* / (1 − *p_ij_*)] = β_*0*_ + β*_1_z_j_* + *u_j_*

where *z_j_* is the value of the social environmental indicator in the *j*th PUMA. We describe the results of these models and our subsequent modeling strategy for the PUMA-level predictors in the Results section. Next, we modeled individual predictors of probability of dying before transport:

Equation 3

log [*p_ij_* / (1 − *p_ij_*)] = β_*0*_ + β*_1_ age 25-49_ij_* + β*_2_ age 50-59_ij_* + β*_3_ age 60-64_ij_* + β*_4_ age 65-69_ij_ + *β*_5_ male_ij_* + β*_6_ white_ij _* + β*_7_ unmarried_ij_* + β*_8_ nocollege_ij_* + *u_j_*

Our final model included individual predictors and 1 PUMA-level predictor:

Equation 4

log [*p_ij_* / (1 − *p_ij_*)] = [equation 3] + β*_9_density_j_*

where density is a dichotomous indicator variable, the derivation of which is described in the Results section.

The intra-class correlations (on the logit scale) reveal the percentage of total variance at the small-area level: ρ = *σ_u_^2^* / (*σ_u_^2^* + *σ_e_^2^*), where *σ_e_^2^* is the variance of the standard logistic distribution, estimated by π^2^ / 3 = 3.29. Odds ratios (ORs) and estimated probabilities were obtained from the GLIMMIX procedure.

## Results

Our study population consisted of 2,570 people aged 25 to 69 years who died of cardiac events (Table 1). Most were men (70%), white (87%), and had at least a high school education (80%). AMI was the most common underlying cause of death (75%), followed by ID causes. The most common place of death was a hospital, followed by home and hospital emergency room. Overall, 35% of these decedents died without transport.

We examined the distributions of social environmental indicators of population density and low social cohesion across the 20 PUMAs in our study area (Table 2). A minority lived alone (range, 8% to 19%) and most had lived at their current residence for fewer than 5 years. There was marked variation in the percentage of housing units sited on 1 acre or more of land and in the percentage that were single-family detached homes. The variation in total land area of the PUMAs (range, 32 square miles to 589 square miles) reflects population density, because PUMAs with smaller populations are larger in land area.

In the crude analysis, we observed geographic disparity in the percentage of persons who died from cardiac events without transport (Figure and Table 3). Multilevel statistical modeling provided a rigorous approach to partitioning the variance between contextual and compositional effects. The null model revealed that little of the variance in our data was found at the small-area level (0.9%). Furthermore, none of the random intercept parameter estimates for individual PUMAs was significant (Table 3). Therefore, regression estimates for the social environmental indicators were obtained by modeling each variable separately as a fixed effect, with the random intercept effects for small areas (data not shown). Three small-area indicators were significant in these models: total land area, percentage of housing units on 1 or more acres of land, and percentage of the study population living alone. We then modeled these small-area–level effects 2 at a time and found that percentage of housing units on 1 or more acres, an indicator of low population density, was the only remaining predictor. When we examined the underlying distribution of this variable, we found that it was strongly bimodal: 15 PUMAs had less than 9% of housing units on 1 or more acres, and 5 PUMAs had 23% or more. We created a new variable called density to capture this bimodal distribution.

**Figure.**
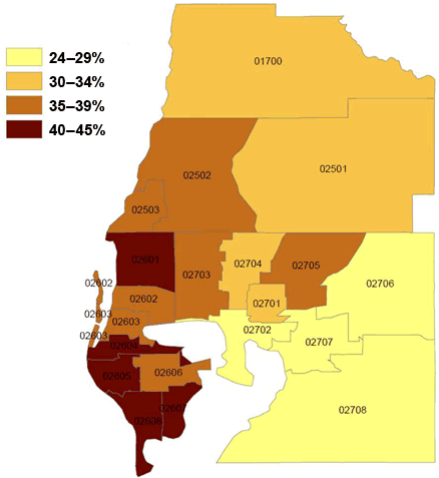
Small-area variation in percentage of people in the Tampa, Florida, metropolitan statistical area who died from cardiac events without transport to a hospital (N = 2,570), 1998-2002. Public use microdata areas from US Census Bureau's public use microdata 5% sample (14).

**Table:** 

**Public Use Microdata Areas**	**Percentage Without Transport**
01700	30.6
02501	32.7
02502	34.6
02503	38.2
02601	41.3
02602	35.6
02603	36.0
02604	39.9
02605	44.6
02606	38.5
02607	41.8
02608	40.7
02701	33.1
02702	25.4
02703	36.0
02704	30.3
02705	35.9
02706	23.5
02707	28.9
02708	26.9

Several decedent characteristics were significant predictors of probability of dying before transport, including younger age, non-Hispanic white race, and being unmarried at the time of death (OR, 2.16; 95% confidence interval [CI], 1.82-2.56) (Table 4). Educational attainment and sex showed no significant association. Our final model included the PUMA-level fixed effect for population density, which was found to be slightly protective against dying without transport (OR, 0.81; 95% CI, 0.67-1.01; *P* = .06).

## Discussion

Multilevel statistical modeling revealed that almost none of the variance in our data was found at the geographic (small-area) level. Rather, the small-area–level variation we observed was almost completely due to compositional effects. This finding contradicted our a priori hypothesis that low population density and low social cohesion would be important in determining the transport status of people who died from cardiac events. Instead, we found that being unmarried, a proxy of living alone and perhaps of a lack of social support, was the strongest predictor of dying without transport for cardiac decedents. Previous research findings have consistently shown heart disease death rates are higher for unmarried people than for married people (18-22), which could be due to higher incidence of heart disease (23,24), higher case fatality (21), or both. Our study suggests that at least some portion of this excess is due to higher case fatality: unmarried people who live alone may have impaired access to life-saving EMS. Future studies could directly test this hypothesis through linked EMS and patient outcome data.

Our results showing a higher probability of whites dying with no transport compared with blacks and Hispanics are consistent with earlier national findings (3). This result is surprising from the perspective of patient delays in seeking treatment, because several studies have shown that blacks in particular delay seeking treatment longer than whites. However, our findings could result from unmeasured differences in household composition between whites and other racial/ethnic populations. National census data reveal that racial/ethnic minorities are less likely to live alone than whites and are more likely to live in extended family households (25,26).

The REACT trial found that people who lived alone were more likely than others to use EMS for transportation to a hospital, perhaps reflecting a lack of other transportation options (27). However, the study's respondents were much more likely to call EMS when they were bystanders to the cardiac event of a stranger than when they were personally experiencing cardiac symptoms (27). Although the symptoms experienced in cardiac events often differ from typical and expected symptoms (28), the incongruity in symptoms does not explain delays in seeking treatment (29). In a study of rural residents, the most important predictors of decision time to seeking treatment were lack of ability to carry out normal activities and extent of anxiety (29).

Notable strengths of our study include accurate geocoding, specificity of cardiac causes of death, and the use of multilevel modeling. In addition, the validity of death certificate data on Hispanic ethnicity, black race, white race, and educational attainment has been high (30-32). We included both suspected and definite acute cardiac deaths to avoid selection bias, because many out-of-hospital deaths (eg, nontransported) lack accurate cause-of-death coding. However, it is likely that we also introduced some degree of misclassification, as some of the ID deaths may not have been cardiac in origin. Furthermore, our use of death certificate data entails other limitations. Primarily, we did not have access to data for a true denominator of all acute cardiac events that occurred in our study area during the study period (ie, a study population that included both decedents and survivors). Unlike the case for cancer, no surveillance system of incident coronary heart disease exists in the United States. In addition, data about decedent medical history and socioeconomic status were not available.

Our study was conducted in a large ethnically and geographically diverse area, which improves the generalizability of our findings. However, none of the rural areas in the Tampa MSA could be considered isolated or remote. Future studies in remote rural areas may well find a stronger effect of geographic and social environmental factors. Furthermore, our study area has well-funded and extensive local EMS provider agencies, which may minimize the barriers of location and distance on transport status once medical aid is requested.

Our study showed that low population density and low population social cohesion were not impediments to transport before cardiac death. It appears that a smaller geographic scale (ie, the household) is the critical level for interventions to reduce delay times for seeking treatment for acute cardiac events. Previous research has shown that the public is more willing to intervene and help a stranger or family member than to seek aid and treatment for self-suffering (27). Interventions are needed to overcome the reluctance of many patients to take action to help themselves. People who are unmarried and live alone are particularly vulnerable to dying from cardiac events without medical aid or witness.

## Figures and Tables

**Table 1:** Characteristics of People Aged 25-69 Years Who Died of Cardiac Events, Tampa, Florida, Metropolitan Statistical Area (MSA), 1998-2002

**Characteristic**	No. of Deaths Without Transport (n = 905) (%)	No. of Deaths After Transport (n = 1,665) (%)	Total (N = 2,570) (%)
**Age at death, y**			
25-49	185 (20.4)	229 (13.8)	414 (16.1)
50-59	275 (30.4)	453 (27.2)	728 (28.3)
60-64	205 (22.7)	387 (23.2)	592 (23.0)
65-69	240 (26.5)	596 (35.8)	836 (32.5)
**Sex**			
Men	642 (70.9)	1,156 (69.4)	1,798 (70.0)
Women	263 (29.1)	509 (30.6)	772 (30.0)
**Race and Hispanic ethnicity**			
White, non-Hispanic	805 (89.0)	1,432 (86.0)	2,237 (87.0)
Black, non-Hispanic	70 ( 7.7)	160 (9.6)	230 (9.0)
Hispanic	30 (3.3)	73 (4.4)	103 (4.0)
**Educational attainment**			
Not a high school graduate	168 (18.6)	355 (21.3)	523 (20.4)
High school graduate	447 (49.4)	811 (48.7)	1,258 (49.0)
Some college	174 (19.2)	289 (17.4)	463 (18.0)
College graduate	116 (12.8)	210 (12.6)	326 (12.7)
**Marital status at time of death**			
Married	377 (41.7)	1,014 (60.9)	1,391 (54.1)
Unmarried	528 (58.3)	651 (39.1)	1,179 (45.9)
**Underlying cause on death certificate**			
Acute myocardial infarction	548 (60.6)	1,387 (83.3)	1,935 (75.3)
Cardiac arrest and dysrhythmias	31 (3.4)	104 (6.3)	135 (5.3)
Cardiovascular disease, unspecified	70 (7.7)	71 (4.3)	141 (5.5)
Ill-defined and unknown causes	256 (28.3)	103 (6.2)	359 (14.0)
**Place of death**			
Hospital inpatient	0	1,035 (62.2)	1,035 (40.3)
Hospital emergency room or outpatient	0	608 (36.5)	608 (23.7)
Hospital, unknown inpatient or outpatient status	0	10 (0.6)	10 (0.4)
Dead on arrival	0	12 (0.7)	12 (0.5)
Decedent's home	797 (88.1)	0	797 (31.0)
Other specified location	107 (11.8)	0	107 (4.2)
Unknown	1 (0.1)	0	1 (0.0)
**Area of residence^a^**			
Low population density	220 (24.3)	497 (29.9)	717 (27.9)
High population density	685 (75.7)	1,168 (70.2)	1,853 (72.1)

a Based on land area, in square miles, in the public use microdata area from US Census Bureau's public use microdata 5% sample (14). *Low* was defined as 23% or more housing units on 1 or more acres, and *high* was defined as less than 9% of housing units on 1 or more acres.

**Table 2:** Social Environment Indicators for Small Areas^a^ (n = 20), Tampa, Florida, Metropolitan Statistical Area, 2000

**Indicators**	Mean	Median	Minimum	Maximum
**Social cohesion**				
Population at risk who lived alone, %	13.4	13.6	7.7	19.3
Population at risk who had lived at same address for <5 y, %	57.2	56.6	49.5	72.6
Population at risk who had lived at same address for >10 y, %	26.8	26.7	15.6	33.6
**Population density**				
Area, square miles	167	93	32	589
Housing units on ≥1 acre of land, %	10.6	5.3	2.7	34.1
Housing units that were single-family homes, %	66.9	67.9	43.5	80.2

a Based on land area, in square miles, in the public use microdata area from the US Census Bureau's public use microdata 5% sample (14).

**Table 3:** Small Area^a^ Variation in Percentage of People Who Died of Cardiac Events Without Transport to a Hospital (N = 2,570), Tampa, Florida, Metropolitan Statistical Area, 1998-2002

**PUMA**	No. of Deaths	Percent Without Transport	Covariance Parameter Estimate From Null Model	*P* Value
01700	193	30.6	−0.1075	0.36
02501	156	32.7	−0.0497	0.68
02502	188	34.6	−0.0089	0.94
02503	165	38.2	0.0730	0.54
02601	80	41.3	0.0938	0.50
02602	104	35.6	0.0112	0.93
02603	125	36.0	0.0207	0.87
02604	153	39.9	0.1067	0.38
02605	139	44.6	0.1980	0.11
02606	156	38.5	0.0771	0.53
02607	194	41.8	0.1642	0.15
02608	155	40.7	0.1241	0.31
02701	136	33.1	−0.0381	0.76
02702	122	25.4	−0.1868	0.15
02703	75	36.0	0.0151	0.91
02704	99	30.3	−0.0800	0.55
02705	53	35.9	0.0101	0.94
02706	102	23.5	−0.2014	0.13
02707	97	28.9	−0.1033	0.44
02708	78	26.9	−0.1186	0.40

Abbreviation: PUMA, public use microdata area.

a Based on land area, in square miles, in the PUMA from the US Census Bureau's public use microdata 5% sample (14).

**Table 4:** Multilevel Modeling Results for Individual and Area Characteristics as Predictors of Death Without Transport to a Hospital Among People Who Died of Cardiac Events (N = 2,570), Tampa, Florida, Metropolitan Statistical Area, 1998-2002

**Characteristic**	Null (Random Effects Only) Model, OR (95% CI)	Model Including Decedent Characteristics, OR (95% CI)	Model Including Decedent Characteristics and Area of Residence, OR (95% CI)
**Age, y**			
25-49		1.83 (1.42-2.35)	1.82 (1.42-2.35)
50-59		1.45 (1.17-1.80)	1.45 (1.17-1.80)
60-64		1.34 (1.06-1.69)	1.34 (1.06-1.68)
65-69		1 [Reference]	1 [Reference]
**Sex**			
Men		1.11 (0.92-1.34)	1.11 (0.92-1.34)
Women		1 [Reference]	1 [Reference]
**Race/ethnicity**			
White		1.43 (1.10-1.87)	1.49 (1.15-1.94)
Black or Hispanic		1 [Reference]	1 [Reference]
**Marital status**			
Unmarried		2.16 (1.82-2.56)	2.13 (1.79-2.52)
Married		1 [Reference]	1 [Reference]
**Education level**			
No college degree		0.99 (0.77-1.27)	1.01 (0.79-1.31)
College degree		1 [Reference]	1 [Reference]
**Area of residence^a^**			
Low population density			0.81 (0.67-1.01)
High population density			1 [Reference]
**PUMA variance**	0.0283	0.0118	0.0062
**Total variance at PUMA level, %**	0.9%	0.4%	0.2%

Abbreviations: OR, odds ratio; CI, confidence interval; PUMA, public use microdata area.

a Based on land area, in square miles, in the PUMA from US Census Bureau's public use microdata 5% sample (14). *Low* was defined as 23% or more housing units on 1 or more acres, and *high* was defined as less than 9% of housing units on 1 or more acres.
